# Motor Function Evaluation of Hemiplegic Upper-Extremities Using Data Fusion from Wearable Inertial and Surface EMG Sensors

**DOI:** 10.3390/s17030582

**Published:** 2017-03-13

**Authors:** Yanran Li, Xu Zhang, Yanan Gong, Ying Cheng, Xiaoping Gao, Xiang Chen

**Affiliations:** 1 Department of Electronic Science and Technology, University of Science and Technology of China, Hefei 230027, China; liyanran@mail.ustc.edu.cn (Y.L.); yngong@mail.ustc.edu.cn (Y.G.); xch@ustc.edu.cn (X.C.); 2 Department of Rehabilitation Medicine at the First Affiliated Hospital of Anhui Medical University, Hefei 230022, China; chengyingy@hotmail.com (Y.C.); gxp678@163.com (X.G.)

**Keywords:** electromyography, inertial measurement unit, motor function evaluation, stroke

## Abstract

Quantitative evaluation of motor function is of great demand for monitoring clinical outcome of applied interventions and further guiding the establishment of therapeutic protocol. This study proposes a novel framework for evaluating upper limb motor function based on data fusion from inertial measurement units (IMUs) and surface electromyography (EMG) sensors. With wearable sensors worn on the tested upper limbs, subjects were asked to perform eleven straightforward, specifically designed canonical upper-limb functional tasks. A series of machine learning algorithms were applied to the recorded motion data to produce evaluation indicators, which is able to reflect the level of upper-limb motor function abnormality. Sixteen healthy subjects and eighteen stroke subjects with substantial hemiparesis were recruited in the experiment. The combined IMU and EMG data yielded superior performance over the IMU data alone and the EMG data alone, in terms of decreased normal data variation rate (NDVR) and improved determination coefficient (DC) from a regression analysis between the derived indicator and routine clinical assessment score. Three common unsupervised learning algorithms achieved comparable performance with NDVR around 10% and strong DC around 0.85. By contrast, the use of a supervised algorithm was able to dramatically decrease the NDVR to 6.55%. With the proposed framework, all the produced indicators demonstrated high agreement with the routine clinical assessment scale, indicating their capability of assessing upper-limb motor functions. This study offers a feasible solution to motor function assessment in an objective and quantitative manner, especially suitable for home and community use.

## 1. Introduction

Motor function impairment is the sequelae of lots of neuromuscular diseases or injuries, such as strokes, spinal cord injuries, cerebral palsy and some others. It may significantly reduce self-care ability and quality of life for the patients, and therefore represent a heavy burden for their family and society [1]. A variety of therapeutic approaches have been developed for the clinical management and treatment of motor impairments, where assessment of motor function is always involved. The motor function evaluation is able not only to quantify the degree of motor dysfunction in patients but also to measure the clinical outcome of the applied intervention. It further offers important guidance for clinicians to establish rehabilitation protocols for individual patients. Therefore, the clinical evaluation of motor function is considered as a prerequisite to the development of effective approaches towards enhanced therapeutic effect [2].

Generally, the use of standardized assessment scales serves as the clinical routine to measure the motor function of patients by clinicians through their own visual sense or patients’ self-report [2]. However, subjectivity and low-sensitivity are two main shortcomings of applying this routine way. Multiple clinicians may give different scores for the same patients. Besides, human visual system might not notice some tiny changes in the motor function of a patient. Sometimes, such assessment approaches may be verbose. For instance, it would take a long time to use the Fugl-Meyer (FM) assessment scale for rating the level of motor function because it consists of 50 items examining mainly fine motor skills [2]. An added shortcoming is that the evaluation needs to be operated by professionals, indicating its inability to be implemented anytime and anywhere, especially for home or community use. Therefore, there have been increasing demands for developing effective approaches to evaluate motor function in an objective and convenient way. These approaches would have motor function measurable in numerical terms by applying motion capture technique, which could facilitate the evaluation approach and enable monitoring for outcomes of clinical interventions during the rehabilitation process.

The key to achieve the quantitative evaluation of motor function is to sense the motion of human body so as to analyze motion abnormalities. In terms of the sensing technology employed to capture motion data, a variety of reported approaches can be summarized into several categories, including techniques based on computer vision (i.e., cameras), inertial sensors, pressure sensors and electromyography (EMG) sensors, respectively. The computer vision-based techniques interpret motions by the means of acquiring, processing, analyzing, and understanding images of human body movements [3,4,5,6,7]. For example, the Vicon system consisting of multiple infrared high-speed cameras and an associated software can be used to capture the motion data [3], for gait analysis and even the motor function evaluation [4,5]. However, site-specific constraint limits wide applications of the computer vision-based techniques. The inertial sensors such as accelerometer, gyroscope, or combination of both termed as inertial measurement unit (IMU), can capture kinematic information about the body movement when placed over appropriate body parts [8,9,10]. Some studies reported successful applications of the accelerometers in monitoring the daily living ability of stoke survivors [11,12,13]. However, in these studies, participants needed to wear accelerometers for a long time like 24 h or even 3 days during the active/inactive periods. Patel et al. [14] purposefully selected eight tasks from the Function Ability Scale and gave each task an estimated score through pattern recognition analyses of the accelerometer data. Gubbi et al. [15] developed an approach to calculate an index equivalent to the National Institute of Health Stroke Score (NIHSS) motor index of stroke patients by measuring the acceleration of the arms. Pressure sensor can be used to offer supplementary kinematic information in terms of the imposed pressure. It has been already used in many studies involving gait analysis [16]. The surface EMG sensor is able to measure electrical potentials generated from muscle contractions in a nonintrusive way [17]. Therefore, the EMG-based techniques have been widely used for context awareness [17], motor control analysis [18], rehabilitation training [19], and motion pattern recognition and interaction [20,21,22,23,24,25].

Recently, a series of sensors like pressure sensors, IMUs and EMG sensors have been embedded in many smart devices with capability of movement sensing due to their low-cost, wearable and self-contained features. Meanwhile, multi-source data fusion technique has become a very popular research topic in many fields, such as gait analysis [9,16], motion pattern recognition [20,21,22,23,24,25], and movement monitoring [26,27,28]. The combination of both IMU and EMG sensor has been found to take advantage of complementary information that help enhance performance of gestural control [20,21,22,23,24,25] and motor function assessment for people with disabilities [27,28]. However, the development of motor function assessment relying on fusion of wearable sensors is still insufficient.

In this paper, a novel framework for upper-limb motor function assessment was proposed based on information fusion of wearable IMU and surface EMG sensors. A set of 11 canonical tasks was specifically designed for subjects to perform during the test, and meanwhile a series of unsupervised and supervised machine learning algorithms were accordingly applied to the recorded motion data. Given the data from healthy subjects, the normal pattern of the task performance was established as standard reference. Therefore, for a given subject, the upper limb motor function was quantified by evaluation indicators representing the degree of motor abnormality with respect to that normal reference. The study can be regarded as an evolution of our recently reported gestural sensing technology [20,21,22,23] using combined IMU and EMG sensors toward motor function evaluation as well as clinical outcome measurement. The feasibility of the proposed framework was indeed demonstrated with data from hemi-paretic stroke survivors.

## 2. Materials and Methods

### 2.1. Sensing Devices

**Figure 1:**
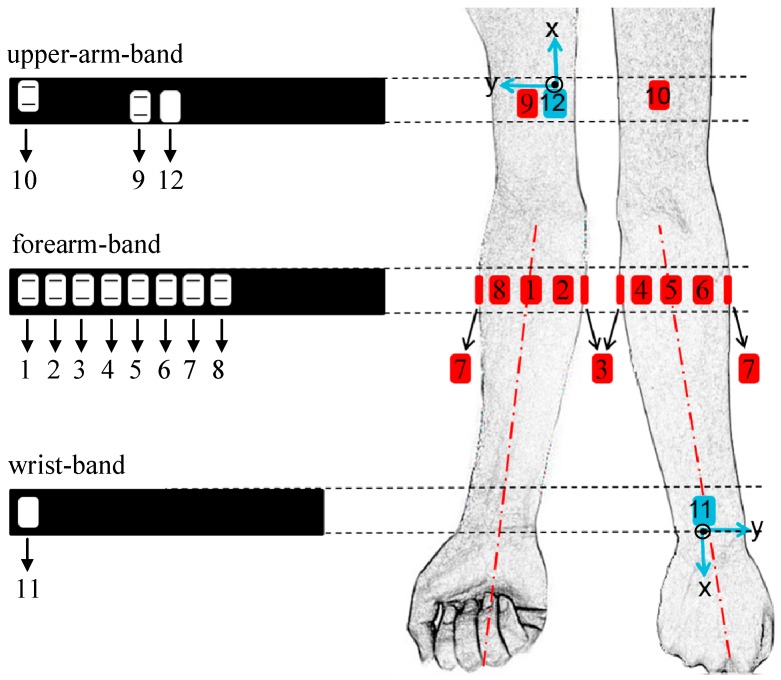
Schematic of the placement and orientation of the sensors in the experiment. The right upper limb is taken as an example to illustrate here. The red ones stand for EMG sensors, and the blue ones stand for IMUs. IMU’s z-axis is perpendicular outside to the plan.

In order to capture upper-limb movements, a home-made sensing system consisting of two IMUs and 10 surface EMG sensors in total was used for data collection in this study. In the system, multiple separate sensing devices were designed in a wrist-band or arm-band formation to ensure its wearability. In this study, one wrist-band and two arm-bands (placed over both upper-arm and forearm) were employed for sensing movements of the upper-limb including subtle fingers, wrist, elbow and shoulder, as shown in Figure 1, where the right arm was used as an example. One IMU (MPU-9250, InvenSense, San Jose, CA, USA; including a 3-axis ACC and a 3-axis GYRO, denoted as IMU1) was embedded in the wristband, located at the middle of the back of the forearm. A round reference electrode (Dermatrode; American Imex, Irvine, CA, USA) was also placed within the wristband over the front side of the forearm. The forearm band consisted of eight surface EMG sensors, evenly distributed around the maximal circumference of the forearm cross-section. The EMG sensor #1, #3, #5 and #7 were place over the central line of the anterior side, the ulnar side, the central line of the posterior side and the radial side of the forearm. For the upper-arm band, two EMG sensors targeting at the biceps brachii and the triceps brachii muscles respectively were embedded in the inner side of a stretchable belt, while an IMU (denoted as IMU2) attached on the opposite side of the belt to the surface EMG sensor over the biceps. The stretchability of these bands ensured the sensors remained firmly fixed at their targeted positions. Besides, the sensor-placement on the left was symmetrical with that on the right one. Each surface EMG sensor included two parallel bar-shaped dry electrodes (1 mm × 10 mm, with 10 mm center-to-center distance) to constitute a single-differential recording channel. In this system, the signal of each EMG channel was amplified with a gain of 600 in total and further digitized by a 12-bit A/D converter (ADS1198, Texas Instruments, Dallas, TX, USA). The sampling rate of each EMG channel was 1000 Hz. The IMU was able to produce digitalized data with a sampling rate of 100 Hz for each axis. All the recorded data were wirelessly transmitted to a computer. This study used a laptop computer to store the data into its hard disk for off-line analysis in a Matlab environment (version 2014a, The Mathworks Inc., Natick, MA, USA).

### 2.2. Subjects

Sixteen healthy subjects (6 females and 10 males, age: 24–63, averaged 36.25 ± 15.19 years) and eighteen stroke subjects with substantial hemiparesis (7 females and 11 males, age: 30–81, averaged 55.28 ± 12.25 years) were recruited in the data collection experiment, which was approved by local ethic review boards. The informed consent was obtained from all subjects prior to their participation. All healthy subjects had no history of joint or neurological disorders or injuries. All stroke subjects did not suffer from evident muscle atrophy on the impaired forearm as compared with the unimpaired forearm, and had no cognitive difficulties. They were at the third or later stage in Brunnstrom’s Approach [29], being clinically stable to participate into the experiment. The paretic arm was tested for each stroke subject, and each healthy subject was tested unilaterally via a random selection. Moreover, for the stoke subjects, the motor function of the tested arm was reported through the upper extremity component of the Fugl-Meyer assessment scale (FMUE) by a clinician right before the experiment. There are 33 items in total in the FMUE scale. Each item is scored on a three-level scale ranging from 0 (indicating that the assignment cannot be performed at all) to 2 (indicating that the assignment can be performed in an ideal way), resulting in the total FMUE score of 66. Evidently, each healthy subject had a full score of 66 by FMUE evaluation. The demographic information of all hemiplegic subjects can be found in Table 1.

### 2.3. Design of Standard Testing Tasks

**Figure 2:**
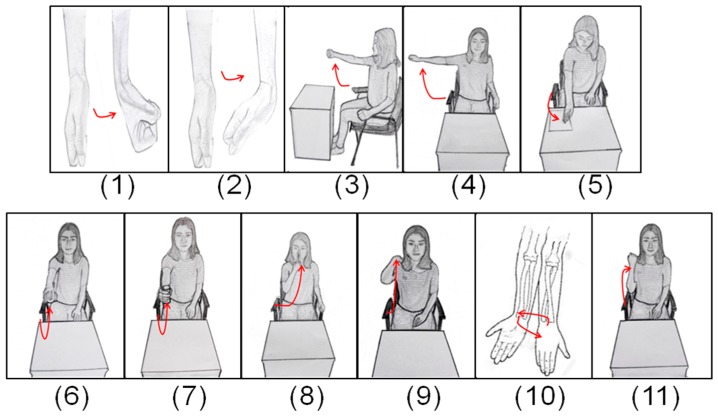
Sketch map of the canonical tasks (taking the right upper limb as an example): (**1**) wrist flexion; (**2**) wrist extension; (**3**) shoulder flexion to 90°, elbow at 0°; (**4**) shoulder abduction to 90°, elbow at 0° and forearm pronated; (**5**) flip a piece of paper; (**6**) fetch and hold a ball put on the table initially and keep shoulder flexion to 90°, elbow at 0° and palm down; (**7**) fetch and hold a cylindrical roll put on the table initially and keep shoulder flexion to 90°, elbow at 0°and palm towards the body; (**8**) finger to nose; (**9**) touch the back of the shoulder; (**10**) keep shoulder at 0°, elbow at 90° and palm down, then do supination/pronation of the forearm for twice; (**11**) flex elbow three times as fast as possible.

We purposely designed 11 canonical tasks (denoted as TASK1–11) for upper-limb motor function evaluation as shown in Figure 2. They are composite tasks derived from individual items in FMUE scale and some activities frequently used in daily life. These tasks involved general movements of the major upper-limb joints, including the shoulder, elbow, wrist, and finger joints, being able to generally reflect gross motor function of the entire upper-limb. Another advantage of using these 11 tasks other than the 33 tasks in the original FMUE scale is to significantly shorten time cost during the test, thus being suitable for mobile testing with wearable sensors. Specifically, TASKs 1, 2 and 9 were used to examine the motor ability associated with the wrist flexion/extension, pronation/supination. TASKs 3–8 were selected from relatively complicated activities in ADLs, which comprehensively examined the functional movement of the upper limb. In addition, TASK 10 was primarily used to examined tremor and dysmetria, and TASK 11 to examine the ability to perform a common elbow flexion/extension task at a high speed. It is worth noting that each designed canonical task could be highly associated with one or some items in the FMUE scale. For instance, the completion of TASK 6 generally involved four FMUE items in terms of examined motor function, thus being able to yield a FMUE score from 0 to 8 to account for degree of the task performance. According to the original FMUE scale, the full scores associated with all individual tasks (TASK1–11) were summarized as being 2, 2, 2, 2, 6, 8, 8, 6, 2, 2, and 2, respectively. Such information was used to examine the availability of evaluation results from the proposed computerized approach in the following analyses.

### 2.4. Experimental Protocol

In the experiment, each subject was seated upright in a comfortable chair, with shoulder abduction at almost 0 degrees, elbow flexion at approximately 90 degrees, and the tested forearm placed on the height-adjustable arm of the chair. After the sensing devices were securely worn on the tested arm, the subject was asked to be fully relaxed. This was regarded as the neutral position of the tested arm during following task performance. For each subject, the testing experiment consisted of 11 trials corresponding to the 11 tasks performed in a random order. In each trial, the subject was asked to implement one task with three repetitions under the guidance of the experimenter. The healthy subjects always performed each task (except the speed-sensitive TASK 11) at a comfortable and common daily speed, whereas the stroke subjects were asked and encouraged to try their best to complete the task so as to truly reflect their motor ability. After completing one task performance, the tested arm needed to return to its neutral position, with a time interval of 10 s for the next repetition. There was also a rest period of at least 30 s between consecutive trials to avoid the subject’s mental or muscular fatigue. For the stroke subjects, a longer rest period was allowed. Besides, an expert clinician evaluated motor function of the hemiplegic upper-limb using the FMUE scale right after the above testing experiment.

### 2.5. Data Analysis

**Figure 3:**
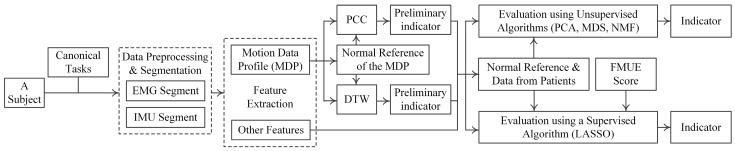
Flow chart of data analysis. PCA: principal component analysis; MDS: multidimensional scaling; NMF: non-negative matrix factorization; LASSO: least absolute shrinkage and selection operator.

Figure 3 shows the data analysis procedure of the proposed framework. The entire evaluation procedure was introduced below: (1) Preprocess data and select segments corresponding to task performance; (2) extract features from each data segment; (3) perform preliminary evaluation using Pearson’s correlation coefficient (PCC) and dynamic time wrapping (DTW); (4) producing evaluation indicators using unsupervised algorithms or a supervised algorithm. Details concerning the data analysis procedure and implementation of the algorithm are provided below.

#### 2.5.1. Data Preprocessing and Segmentation

In order to eliminate potential low-frequency motion artifacts and high-frequency interferences, the raw sEMG signals were band-pass filtered (Butterworth filter, 2nd order) within 20–500 Hz, and the IMU signal was low-pass filtered (Butterworth filter, 2nd order) with a cut-off frequency of 20 Hz.

**Figure 4:**
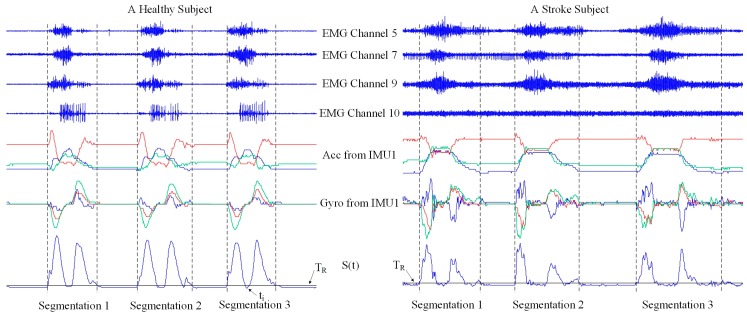
Illustration of the data segmentation process when representative data channels are used as examples from a healthy subject and a stroke subject performing TASK 8, respectively. For each subject, there are three repetitions of task performance. The black solid line represents the threshold T_R_.

After the noise reduction, the recorded data were segmented by detecting both onset and offset of each task repetition. Our previous studies [20,21] successfully utilized EMG amplitude/energy from neurologically intact muscles of gesturers and signers for data segmentation by taking advantage of evident and consistent muscle activities during task performance. In this study, however, it was found that evident EMG activities were likely to present after task performance for stroke subjects due to their inability to fully relax as a result of impaired motor control. With this regard, we intentionally chose the data from both gyroscopes for data segmentation. Suppose that 3 axes of gyroscope signals are x_1_, y_1_, z_1_ for the IMU1 and x_2_, y_2_, z_2_ for the IMU2, respectively. The instantaneous summation of vector magnitudes from both gyroscopes was computed at each moment according to Equation (1). Then a magnitude thresholding approach was performed, where the threshold T_R_ was set to be 3 degrees per second through many pretests. The onset time was determined to be the moment when S(t) rose over the threshold, while the moment when S(t) fell below and its following 2-s signals were kept below the threshold indicated the offset time. If S(t) momentarily fell down below the threshold within 2 s, the data within an entire task performance procedure could not be interrupted. Finally, the determined onset and offset were applied to all data channels (consisting of 10 EMG channels, 6 accelerometer axes and 6 gyroscope axes in total) to select a data segment corresponding to each task repetition. Figure 4 illustrates the data segmentation process in detail. The following feature extraction and motor function evaluation analyses were performed on these selected data segments:

(1) $$S\left(t\right)=\sqrt{x_{1}^{2}\left(t\right)+y_{1}^{2}\left(t\right)+z_{1}^{2}\left(t\right)}\;+\;\sqrt{x_{2}^{2}\left(t\right)+y_{2}^{2}\left(t\right)+z_{2}^{2}\left(t\right)}$$

#### 2.5.2. Feature Extraction

From each data segment, a variety of parameters were extracted as features to characterize the performed task pattern. In this study, the feature extraction process took special considerations of discriminatory information for identifying motor function degrees of different subjects performing a certain task, other than identifying task patterns performed by a certain subject, which has been extensively reported in many previous studies [20,21,22,23]. Therefore, the features examined in this study are introduced and categorized into seven types as follows:

- *Motion data profile (MDP)*: The profile of each data segment is a straightforward representation of the task performance. In order to calculate motion data profile, the recorded data were processed according to sensor type. For each channel of surface EMG signals, a moving average processing was first performed to produce an EMG envelope through calculating mean value of rectified EMG signals within a sliding window with a window length of 256 ms and a window increment of 8 ms. Then, all channel EMG envelopes were simultaneously normalized in amplitude by the maximal value among all envelop values in 10 channels. The 6-axis accelerometer data from two IMUs were normalized in magnitude by its maximal absolute value so as to keep the signals within the range between −1 and +1. The similar process was also applied to the 6-axis gyroscope data as well. Subsequently, the normalized data segment consisting of 10 EMG channels, six accelerometer axes and six gyroscope axes was further normalized in time to 256 sample points, to alleviate time duration variation of task performance. Finally, the motion data profile was produced as a 22 × 256 data matrix for each data segment.
- *Time duration*: The time duration of each data segment was specifically calculated to reflect proficiency of task performance, while such information was not involved in the above MDP due to the normalization process.
- *IMU extremum number*: Within each data segment, the number of local minima and maxima was computed for each axis of both IMUs and then summed up as a feature as well.
- *EMG power distribution*: After the root mean square (RMS) of each surface EMG channel was computed, the percentage of one channel EMG RMS to summation of the RMS values from all 10 channels was subsequently obtained, thus producing a 10-element vector indicating EMG power distribution across channels [30].
- *IMU power distribution*: After the root mean square (RMS) of each axis of accelerometer/ gyroscope was computed, the percentage of a RMS value for one accelerometer/gyroscope axis to summation of the RMS values over all three axes was subsequently obtained, thus producing four (from two accelerometers and two gyroscopes) 3-element vectors indicating movement power distribution across axes.
- *Accelerometer/gyroscope intensity ratio*: At each moment, a magnitude of the 3-axis vector of an accelerometer/gyroscope was computed. After the RMS value of the magnitude time series was calculated for each accelerometer/gyroscope, the ratio of such RMS value from the accelerometer/gyroscope in IMU1 to the RMS value in IMU2 was subsequently obtained as a feature.
- *Mean and maximum value*: A mean value and a maximum value of the magnitude time series for each accelerometer or gyroscope was computed, therefore producing eight features from both accelerometer and both gyroscopes.

#### 2.5.3. Motor Function Evaluation

(1) Preliminary Evaluation

Preliminary evaluation was considered as the first one of two stages of evaluation, which measured similarity in task performance represented by some features between any given subject and a truly normal reference obtained from all healthy people. Given the obtained MDP features that were able to represent the task performance as an example, we explained the data processing procedure in the preliminary evaluation. These MDPs from all the healthy subjects were stored and used as the normal reference for that type of features during the evaluation process. The similarity degree could be evaluated by Pearson’s correlation coefficient (PCC) or by distance calculated via a dynamic time wrapping (DTW) algorithm. The DTW is a nonlinear alignment technique for measuring similarity/distance between two time series which may have different lengths or durations [10]. A greater correlation coefficient or a smaller DTW distance means a higher degree of similarity in task performance between the subject and the healthy group.

Given a subject to be tested, there were three repetitions producing three data segments for each individual task. The correlation coefficients between the MDP of one repetition from the subject and all MDPs in the normal reference (generally consisting of 48 samples: three repetitions per subject × 16 healthy subjects) were first calculated, respectively. Among these coefficients, the maximal coefficient was then selected for each repetition. Therefore, the mean of the maximal coefficients was calculated over three repetitions, as a preliminary evaluation indicator (EI) for the task. Similar to the PCC process, we first calculated distances between the MDP of one repetition from the subject and all MDPs in the normal reference via DTW, then selected the minimal distance, and also computed their mean value as a preliminary EI for the task. Please note that these EIs were considered as EI components associated with individual tasks, their direct summation was computed as the EI globally evaluating the motor function of the entire upper limb.

Besides the MDPs, another type of features, namely EMG/IMU power distribution, was also processed in a similar way, where a Euclidean distance was adopted instead of the distance measure using PCC or DTW. Consequently, for each data segment, there were seven indicators/distances (PCC and DTW indicators from the MDP, Euclidean distances from the EMG power distribution and IMU power distribution of two accelerometers and two gyroscopes) in total at the preliminary evaluation stage. Please note that when a healthy subject was considered as the testing subject to evaluate the performance of the preliminary indicators, the subject’s data were excluded from the normal reference. In addition, by producing the preliminary indicators, the effect of data fusion was also investigated using different data sets: EMG data alone, IMU data alone, and the combined data from both kinds of sensors, respectively.

(2) Evaluation Using Unsupervised Machine Learning

In the later stage of evaluation, the seven preliminary indicators/distances were further considered as features. They were concatenated with other features (those features that were not used in the initial stage of evaluation) as an extended vector for each data segment. Subsequently, we applied some unsupervised machine learning algorithms on these features for the fusion of different feature types, with the purpose to produce more advanced EIs. Here the “unsupervised approach” refers to concealment of any known FMUE score during the learning approach.

For each task, assume that $V^{m\times n}$ is a data sample matrix consisting of m feature vectors (*n* dimensions) in total from all subjects, with each row representing an n-dimensional feature vector. In essence, we built a matrix factorization process to produce the EI, as expressed as:

(2) $$V^{m\times n}=W^{m\times s}H^{s\times n}\;V=\left[\begin{array}{c} V_{1}\\ \vdots \\ V_{m} \end{array}\right]=\left[\begin{array}{ccc} V_{11} & \cdots & V_{1n}\\ \vdots & \ddots & \vdots \\ V_{m1} & \cdots & V_{mn} \end{array}\right],\;W=\left[\begin{array}{ccc} W_{11} & \cdots & W_{s1}\\ \vdots & \ddots & \vdots \\ W_{1m} & \cdots & W_{sm} \end{array}\right],\;H=\left[\begin{array}{ccc} H_{11} & \cdots & W_{1n}\\ \vdots & \ddots & \vdots \\ H_{s1} & \cdots & W_{sn} \end{array}\right]$$

where **W** represents the transformed data matrix with feature dimensionality reduced to s, and $H^{-1}$ represents the transformation matrix. We set *s* = 1 in this study, since the produced EI should be a one-dimensional quantitative score. Three commonly used unsupervised algorithms for matrix decomposition are principal component analysis (PCA), multidimensional scaling (MDS), and non-negative matrix factorization (NMF). We examined their applications in this paper, with a brief introduction as follows:

- *PCA algorithm*: PCA is a very popular technique for dimensionality reduction. Given a set of high-dimensional data, PCA aims to find a linear subspace of lower dimension and such a reduced subspace attempts to maintain most of the variability of the data [31]. In the process of factorization, **V** was centralized first to eliminate the influence of dimension. The transformation matrix $H^{-1}$ would be obtained by obtaining the eigenvalue and eigenvector of the covariance matrix of the centralized matrix.
- *MDS algorithm*: MDS is another classical approach that maps the original high dimensional space to a lower dimensional space with an attempt to preserve pairwise distances [31]. In the process of performing the metric MDS, a squared proximity matrix is set, with elements $d_{ij}^{*}$ representing the Euclidean distances between high-dimensional sample *i* and *j* (*i*, *j* = 1, …, *m* and *i* ≠ *j*,). Sammon’s nonlinear mapping criterion was chosen as the goodness-of-fit criterion. It aims to minimize the loss function Stress [32] given in Equation (3), where $d_{ij}$ is the distance between low-dimensional sample *i* and *j*. These distances $d_{ij}$ is initialized to be random values and then updated via a iterative process using rules reported in [32] so as to minimize the Stress:
  (3) $$Stress_{D}\left(V_{1},V_{2},\ldots ,V_{m}\right)=\frac{1}{\sum_{i<j}d_{ij}^{*}}\underset{i<j}{\sum }\frac{{\left(d_{ij}^{*}-d_{ij}\right)}^{2}}{d_{ij}^{*}}$$
- *NMF algorithm*: This method of matrix decomposition has previously and widely been used for muscle synergy analysis [18]. In this paper, NMF was used for dimensionality reduction just like the above two algorithms. In the process of factorization, **W** and **H** were initialized to be random values first, and were updated using rules [18] given in Equation (4):
  (4) $$W_{ij}\leftarrow W_{ij}\frac{{\left(VH^{T}\right)}_{ij}}{{\left(WHH^{T}\right)}_{ij}}\;,\;H_{jk}\leftarrow H_{jk}\frac{{\left(W^{T}V\right)}_{jk}}{{\left(W^{T}WV\right)}_{jk}}\;,W\left(i\right)\leftarrow \frac{W\left(i\right)}{\left\|W\left(i\right)\right\|}\;\forall \;\mathrm{column}\;\prime i\prime ,$$

For each task, the values in the derived $W^{m\times 1}$ was normalized to the full score associated with that task and then were considered as the advanced EI. The direct summation of these EIs over 11 tasks was computed for each subject as a global EI evaluating the motor function of the entire upper limb.

With the dataset used in this study from 16 healthy subjects and 18 stroke subjects, a 34-fold leave-one-out method was employed to evaluate the performance of each unsupervised machine learning algorithm. When one subject was selected for test, the data from the remaining 33 subjects were used in the learning approach. The transformation matrix $H^{-1}$ and the normalization factor given by the learning approach were applied to the input testing data to produce the EI during test for each task and each subject. In addition, mean of the global EIs derived from all healthy subjects was computed and then expanded to 66. The same expansion factor (i.e., 66 divided by the mean value) was applied to EIs from all subjects, so as to conduct a straightforward comparison between the derived EI and the routine FMUE score.

(3) Evaluation using Supervised Machine Learning

In addition to the use of those unsupervised algorithms, the feasibility of a supervised learning algorithm, least absolute shrinkage and selection operator (LASSO), was also explored further in this paper. LASSO is a well-known regression analysis method. It embeds feature selection in the algorithm framework using the 1-norm regularization and is attractive in many applications involving high-dimensional data [33]. Therefore, this algorithm was implemented in order to produce the EI by incorporating more useful information. Given a linear regression with data matrix **V** and the vector of observations *y*, the LASSO solves the L1-penalized regression problem of finding a vector $z={\left(z_{1},\cdots ,z_{m}\right)}^{T}$ to minimize the algebraic expression (5) [34,35]. First, *z* is initialized to be random values and then updated multiple times in iterations through the least angle regression-elastic net algorithm [36,37]. In this paper, the observation values were the FMUE related-item scores and the values in z were the desired EIs mapped from the observation values:

(5) $${\left\|Vz-y\right\|}_{2}^{2}+\lambda {\left\|z\right\|}_{1}$$

Similarly, a global EI was obtained by summing up EIs over all 11 tasks for each subject. A 34-fold leave-one-out method was also employed to evaluate the performance of this algorithm. The EIs from all subjects were scaled by a factor of 66 divided by the mean EI over all healthy subjects in the same way as mentioned above.

(4) The Establishment of Evaluation Criteria

After the global EI was obtained for each subject, the prerequisite to evaluating the motor dysfunction was the establishment of normal range. The mean and standard deviation (SD) of the EIs from all healthy subjects were computed, respectively, and therefore the normal reference range was defined as the interval within the mean ± 1.96 times SD. On this basis, the more deviation of any given subject’s EI from the normal range, the severer degree of motor function abnormality was determined for the subject.

In order to assess the effectiveness of the examined algorithms, two parameters were designed accordingly. One was the normal data variation rate (NDVR) defined as the percentage of 1.96-times SD to the mean value, which described the volatility of the healthy subjects’ EIs. A smaller NDVR value means a better validity. In addition, the linear regression analysis between the FMUE scores and the EIs was applied to obtain the second parameter, determination coefficient (DC). It described consistency between the FMUE scores and the EIs. A higher DC value means a higher consistency.

## 3. Results

**Figure 5:**
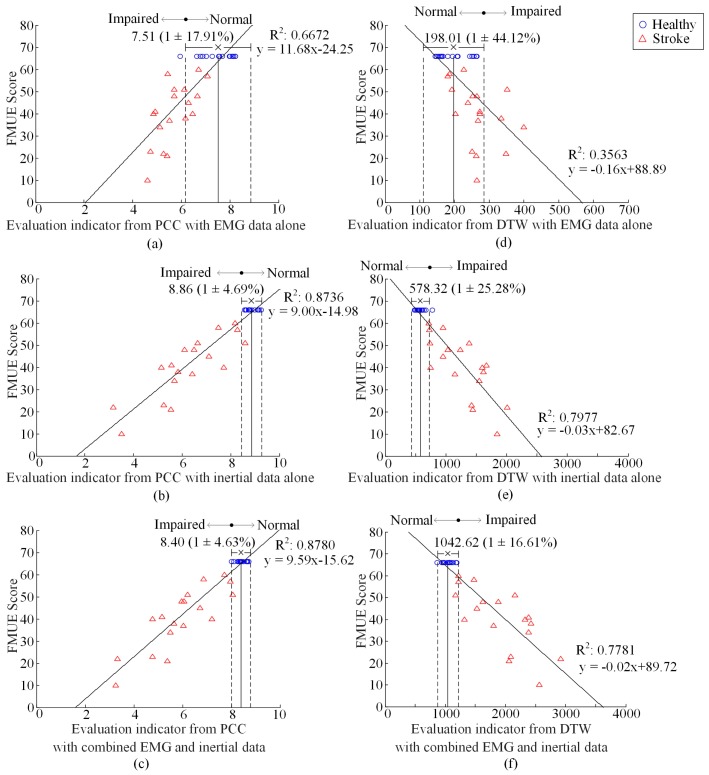
Plot of FMUE score versus the global EI, from PCC (**a**–**c**) and DTW (**d**–**f**) with EMG data alone (**a**,**d**), inertial data alone (**b**,**e**) and combined data (**c**,**f**), respectively. R^2^ reports the coefficient of determination from the linear regression analysis. The ‘×’ and the bar denote the mean and 1.96-times SD of the healthy subjects’ EI. The range between the two dashed lines over the horizontal axis represents the normal range. Circles and triangles represent data from individual healthy and stroke subjects, respectively.

The preliminary EIs for all the examined subjects are summarized in Figure 5, using both PCC and DTW methods, with EMG data alone, IMU data alone, and their combination respectively. Both the derived EI for each subject and its corresponding FMUE score can be expressed as a point in the FMUE-indicator plane, thus forming a scatter plot for all examined subjects. From each scatter plot (each subplot in the Figure 5), it can be found that the points from the healthy subjects are concentrated within a relatively small range along the EI axis. This confirmed our definition of a normal range (two vertical dashed lines) as 1.96 times the SD besides the mean EI (the vertical dot dash line) averaged over all healthy subjects. By defining the normal range, the NDVR was reported to be 17.91%, 4.69% and 4.63%, showing a decreasing trend with the EMG data alone, the IMU data alone and the combined data, when the PCC method was employed. Such a decreasing trend of NDVR was consistently found from 44.12% through 25.28% to 16.61% when using the DTW method. The stroke subjects, by contrast, had their EIs deviated from the normal range. It was also found that for the stroke subjects, the EIs from the PCC or DTW tended to be lower or higher, respectively, than the normal range.

From analyzing the correlation between the FMUE score and the evaluation indicators, the derived DC were found to be dramatically increased, from 0.6672 to 0.8736 using the PCC or from 0.3563 to 0.7977 using the DTW, respectively, with the IMU data alone as compared with the EMG data alone. With the combined data, such a DC was further observed to be increase to 0.8780 using the PCC, whereas use of the DTW lead to a slight decrease of the DC to 0.7781.

**Figure 6:**
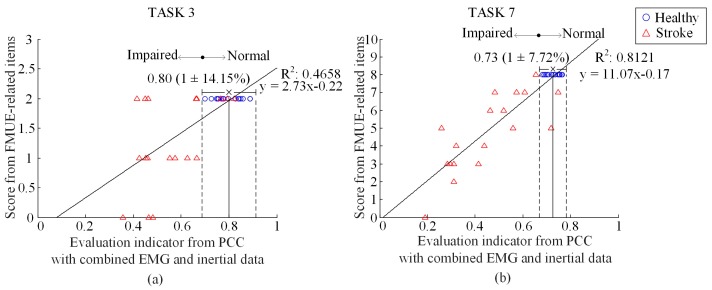
Plot of score of FMUE-related items versus the EI for TASK 3 (**a**) and TASK 7 (**b**). All symbols and lines appear in the same way as illustrated in Figure 5.

The results of motor function evaluation for specific task were also analyzed after assessing the global function of upper limb. Figure 6 reports component EIs from the PCC for the TASK 3 and TASK 7, both used as representative examples, respectively, with combined EMG and IMU data. With similar result representation as Figure 5, it can also be found from the Figure 6 that the healthy subjects have component EIs concentrated within a relatively small range whereas the EIs from the stroke subjects tended to deviated from the normal range. Specifically, the NDVR for TASK 3 and TASK 7 were 14.15% and 7.72%, respectively. By performing regression analysis, the DC reached into 0.4658 for TASK 3 and 0.8121 for TASK 7 respectively.

**Figure 7:**
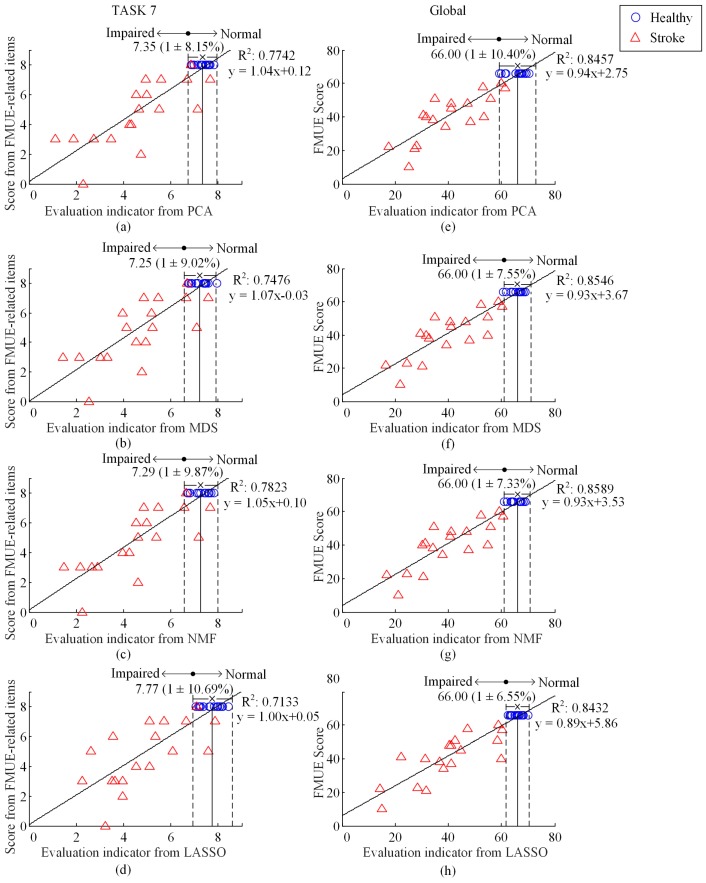
Plot of score of FMUE-related items versus the EI for TASK 7 (**a**–**d**), and plot of FMUE score versus the EI (**e**–**h**), using PCA (**a**,**e**), MDS (**b**,**f**), NMF (**c**,**g**), and LASSO (**d**,**h**), respectively. All symbols and lines appear in the same way as illustrated in Figure 5.

Figure 7 shows component EIs for TASK 7 and the global EIs for all the tasks at the final evaluation stage using PCA, MDF, NMF and LASSO algorithms. Figure 7 also employed the same manner of result representation as previously reported in Figure 5 and Figure 6. By quantitatively evaluating the performance, three unsupervised algorithms PCA, MDS and NMF achieved NDVR values around 9% (i.e., 8.15%, 9.02% or 9.87%, respectively) for TASK 7 and around 10% (i.e., 10.40%, 7.55% or 7.33%, respectively) for the global evaluation including all tasks. When the supervised algorithm LASSO was used, such NDVR values were dramatically increased to 10.69% for TASK 7 and decreased to 6.55% for the global evaluation including all tasks. However, when three unsupervised algorithms and one supervised algorithm were used, comparably DC values were around 0.75 (0.7742, 0.7476, 0.7823, and 0.7133) for TASK7 and 0.85 (0.8457, 0.8546, 0.8589, and 0.8432) for the global evaluation including all tasks were yielded.

## 4. Discussion

This paper presents a framework for quantitative evaluation of upper-extremity motor function based on data fusion from wearable inertial and surface EMG sensors. All these sensors constituted a motion sensing system, which was designed in the form of stretchable bands, so as to be worn around the upper-arm and the forearm conveniently and comfortably. The novelty of the presented framework includes two aspects. (1) A set of 11 canonical tasks representing general functional activities of daily life using the upper-limb were designed by referring to the clinical FMUE scale for motion data collection during the test. As compared with routine FMUE assessment with a complex list of 33 items, implementation of all 11 canonical tasks cost about 20 min for a stroke subject using the hemiplegic upper-limb to complete the testing procedure in our experiment; (2) Accordingly, a quantitative evaluation indicator could be produced by an established model built with appropriate statistical machine learning algorithms. All the examined algorithms were computationally compatible with online processing. When running as a mobile application in the Android environment (Lenovo Tab2, Morrisville, NC, USA), the proposed framework produced a time delay of less than 1 sec for scoring each individual task after its completion. Finally, with the proposed framework employing machine learning methods, this evaluation approach can be conducted without any medical professional, being especially suitable for home and community use.

When the derived EIs were plotted against the FMUE scores for all the subjects, the performance of the proposed evaluation framework can be quantitatively examined by both the NDVR and DC. It was found that the preliminary EI produced via PCC and the EI via supervised LASSO method had two smallest NDVRs of 4.63% and 6.55%, respectively, among all the examined machine learning methods. The small NDVR suggests good ability of the produced EI to quantify normal motor function across multiple healthy subjects with a consistent measure, by overcoming potential effect of individual differences. By contrast, the DTW method yielded the largest NDVR of 16.61%. This suggests that the DTW is likely to measure more subject-specific information beside the common motor function ability, thus compromising its generalization to other healthy subjects. After performing the linear regression analysis, strong DC (>0.8) values were reported between the clinical FMUE score and each of the EIs derived via all examined machine learning methods except DTW. Such a high agreement of the produced EI with the routine clinical scale demonstrate the feasibility of the proposed evaluation framework. This also implies that appropriate selection and definition of the 11 canonical tasks that truly examine the motor function of the entire upper-extremity in this study.

With both the NDVR and DC established for performance evaluation, the effect of EMG and IMU data fusion can be straightforwardly verified. When the PCC was applied on the MDP features to produce a preliminary EI, it was found from the Figure 5 that the use of combined EMG and IMU data led to superior performance in terms of both decreased NDVR and improved DC, over the use of EMG data alone and IMU data alone. When the DTW was used, although the DC did not improve or even slightly decreased with the use of combined data as compared with the IMU data alone, the obvious reduction of NDVR was also found with the combined EMG and IMU data. Such the advantage of EMG and IMU data fusion has been examined by a great many studies in the fields of motion pattern recognition [20,21,22,23,24,25]. In our recent studies, this data fusion technique has been successfully applied to development of rehabilitation training, intelligent gestural interfaces and wearable sign language interpreters [19,20,21,22,23]. The efforts presented in this paper can be regarded as evolution of our technical basis toward a novel application field, the motor function evaluation and rehabilitation following neuromuscular disorders or injuries.

In the proposed framework, the indicator globally evaluating motor function of the entire upper extremity was derived from component EIs evaluating the performance of each individual task. From Figure 6 and Figure 7, it was found that the component EI for any individual task had relatively lower DC value as compared with those of global EI involving all tasks. This may be attributed into inconsistent definition between each canonical task and its manually determined items from the FMUE scale. Another reason for explaining this is the inaccurate and subjective nature of the FMUE scale. Despite of these limitations, the component EIs derived from individual tasks proved the effectiveness of quantifying the motor function purposely examined by an individual task, thus offering essential basis for producing a global EI that enables evaluating the motor function of the entire upper extremity by designing a set of appropriate canonical tasks.

Three unsupervised algorithms, namely PCA, MDS and NMF, yielded comparable performance, with NDVR around 10% and DC slightly beyond 0.8. By contrast, the LASSO algorithm, which conducted the evaluation process in a supervised manner, was able to produce highly centralized EIs from the healthy group, yielding evident NCDR decreased into 6.55%. However, it failed to further improve the DC, as compared with three unsupervised algorithms. Moreover, we surprisingly found that the LASSO algorithm had slightly inferior performance, in terms of larger NDVR and less DC, to the PCC method just producing preliminary EIs. However, the use of LASSO algorithm allowed the global EIs from all stroke subjects to be outside the normal range, whereas one stroke subject’s preliminary EI was within the normal range when the PCC method was used. This indicated improved distinction in motor function deficit of the stroke subjects from the healthy subjects, which was granted by the use of supervised learning.

It should be acknowledged that the relatively small size of subjects draws the main limitation of this study. With limited number of recruited subjects, the work in this paper preliminarily verified the feasibility of the proposed framework for quantitatively evaluating the motor function of hemiplegic upper limb. Another limitation regarding the subject recruitment is the unmatched age range between at the healthy and stroke participants. Although stroke studies generally require age-matched control group, the use of data from healthy subjects with a relatively wider range of ages in this study helps eliminate potential aging effect on the motor function assessment. The next work to be done is to recruit a large number of subjects to systematically evaluate the reliability of this method, so as to further establish the criteria for the evaluation of motor function. Moreover, the involvement of the clinical FMUE score in evaluating the performance of the proposed framework is not sufficiently reasonable due to the well-known issues of the FMUE procedure, such as inaccuracy or subjectiveness. In recent years, some novel and promising techniques such as muscle synergy analysis [18] have been developed for examining neuromuscular changes underlying motor function deficits. The fusion of different aspects information will be conductive to accurately judge the degree and cause of motor dysfunction. All these remain our future work.

## 5. Conclusions

By taking advantage of the wearable property of the combined IMU and surface EMG sensors for motion sensing, this study presents a novel framework for hemiparetic upper-limb motor function evaluation. During the test, a set of 11 canonical upper limb functional tasks was designed for individual subjects to perform using their paretic upper limbs, and meanwhile the motion data were recorded by wearable sensors worn on the tested arm. Within this framework, a series of machine learning algorithms were applied to motion data to produce EIs, which were able to characterize motor abnormality of any given subject by measuring degree of variation in task performance from a standard reference built with healthy group data. Experimental results demonstrated superior performance yielded by the combined IMU and EMG data over the IMU data alone and the EMG data alone, in terms of decreased NDVR and improved DC. Three common unsupervised algorithms achieved comparable performance with NDVR around 10% and strong DC around 0.85. The use of a supervised LASSO algorithm was able to dramatically decrease the NDVR to 6.55%, with improved distinction between the stroke and healthy subjects. All these results verified the effectiveness of the framework proposed. Such a convenient and efficient evaluation system can provide reference for the rehabilitation training, and also offers exciting opportunities for spreading the applications of the proposed technique in community or other places, without the limits of sites or professional skills.

## Figures and Tables

**Table 1:** Demographic information of stroke subjects.

No.	Sex	Height (cm)	Weight (kg)	Paretic Side	Age (Years)	Onset (Days)	FMUE Score	Brunnstrom Stage
1	Male	175	71	Left	72	40	50	4
2	Female	159	48	Right	52	33	58	5
3	Male	181	81	Right	50	11	59	5
4	Male	162	65	Right	58	21	40	4
5	Female	173	66	Right	53	366	37	4
6	Male	176	75	Right	30	457	25	4
7	Male	168	68	Right	61	68	48	4
8	Female	162	49	Left	75	48	40	5
9	Male	176	71	Left	46	49	41	3
10	Male	170	68	Left	69	10	10	3
11	Female	165	55	Left	50	36	21	3
12	Female	158	49	Left	50	51	48	5
13	Male	175	72	Right	51	78	24	3
14	Female	155	45	Right	81	81	51	5
15	Male	178	72	Left	44	225	38	3
16	Female	163	66	Left	51	584	35	4
17	Male	169	73	Left	59	40	57	5
18	Male	175	72	Left	43	71	45	4
